# Clusterin as a potential marker of brain ischemia-reperfusion injury in patients undergoing carotid endarterectomy

**DOI:** 10.1080/03009734.2019.1646359

**Published:** 2019-08-28

**Authors:** Joanna Iłżecka, Marek Iłżecki, Aneta Grabarska, Shawn Dave, Marcin Feldo, Tomasz Zubilewicz

**Affiliations:** a Independent Neurological Rehabilitation Unit, Medical University of Lublin, Lublin, Poland;; b Department of Vascular Surgery and Angiology, Medical University of Lublin, Lublin, Poland;; c Department of Biochemistry and Molecular Biology, Medical University of Lublin, Lublin, Poland;; d University of Oklahoma Health Sciences Center in Oklahoma City, Oklahoma, USA

**Keywords:** Brain injury, carotid endarterectomy, clusterin, potential markers

## Abstract

**Introduction:** Carotid endarterectomy (CEA) is a surgical procedure used in the prevention of ischemic stroke. However, this procedure can cause complications of ischemia-reperfusion injury to the brain. Clusterin (CLU) is a cytoprotective chaperone protein that is released from neurons in response to various neurological injuries. The objective of the study was to report the changes in serum CLU concentrations of patients undergoing CEA.

**Materials and methods:** The study involved 25 patients with severe internal carotid artery stenosis. Serum samples were taken from patients at three different times: within 24 hours preoperatively to CEA, 12 hours postoperatively, and 48 hours postoperatively. Serum CLU concentrations were measured using a commercially available enzyme-linked immunosorbent assay.

**Results:** When compared to concentrations preoperatively, the serum CLU concentration initially decreased during the 12 hours following CEA. However, 48 hours following the procedure there was an increase in the CLU concentration. After statistical analysis, differences were detected in serum CLU concentration between all three recorded measurements (*P* < 0.05).

**Conclusion:** Data from our study indicate that serum CLU concentrations are affected after CEA. We hypothesize that serum CLU concentrations may depend on brain ischemia-reperfusion injury following this surgical procedure.

## Introduction

Carotid endarterectomy (CEA) is a minimally invasive surgical procedure used in the prevention of ischemic stroke. However, this treatment can cause various complications, including embolism resulting in brain ischemia. It was also observed that clamping and declamping of the internal carotid artery during CEA may lead to ischemia-reperfusion injury and brain edema (1,2).

Clusterin (CLU) is also called apolipoprotein J (ApoJ), sulfated glycoprotein-2, secreted glycoprotein gp80, complement lysis inhibitor, testosterone-repressed prostate message 2 (TRPM-2), or complement-associated protein SP40-40. It is found at high concentrations in physiological fluids and is expressed constitutively at variable levels in a wide variety of tissues (3). Expression of the CLU gene (located on chromosome 8p21-p12) leads to the production of the secretory form of this protein found in plasma, cerebrospinal fluid (CSF), and other body fluids (4). CLU is expressed in the human brain by subpopulations of neurons in the neocortex, glial cells in the hippocampus/neocortex, and ependymal cells (5–7). Data from previous literature showed that CLU is a cytoprotective chaperone protein whose concentration is increased in response to a diverse range of stresses including heat, pro-apoptotic insults, oxidative stress, ionizing radiation, and proteotoxicity (8–10). It has been demonstrated that this protein is involved in diverse cellular processes, including apoptosis, cell cycle regulation, DNA repair, and the acquisition of cell resistance against multiple conventional therapies (11). In animal models of ischemic stroke, both neuronal and astroglial cells express high levels of CLU early following ischemic damage (12). CLU binds to a wide range of misfolded client proteins and either sequesters them into stable, soluble complexes or inhibits the formation and accumulation of toxic amyloid assemblies (13,14). This glycoprotein can also inhibit protein aggregation (15). According to Gregory et al. (16), CLU protects neurons against intracellular proteotoxicity. CLU is also a chaperone protein that has the ability to bind to a wide array of physiological ligands involved in the pathogenesis of Alzheimer’s disease. One of these ligands is β-amyloid, for which clusterin mediates clearance from the brain through the blood–brain barrier to the peripheral circulation via megalin (5,17). Moreover, in the peripheral circulation, CLU may prevent low-density lipoprotein (LDL) oxidation by the arterial wall endothelium (18). It was revealed that CLU also exists as a nuclear, unglycosylated 60-kDa protein (19). According to Leskov et al. (20), nuclear CLU can promote cell death.

On the other hand, accumulation of CLU in the cytosol promotes cell survival. Nizard et al. (19) showed stress-induced translocation of CLU from the endoplasmic reticulum to the cytosol. Additionally, this glycoprotein can prevent apoptosis by antagonizing Bax, therefore preventing mitochondrial release of cytochrome C and consequent caspase activation (21). Pereira et al. (22) described protective molecular mechanisms of CLU against apoptosis; according to the authors, CLU can act directly or indirectly on apoptosis by regulating several intracellular pathways. These pathways include: the oxidant and inflammatory program, insulin growth factor 1 (IGF-1) pathway, KU70/BCL-2-associated X protein (BAX) pathway, tumor necrosis factor alpha (TNF-α) pathway, BCL-2 antagonist of cell death (BAD) pathway, and mitogen-activated protein kinase (MAPK) pathway. According to Zinkie et al. (23), the regulation and biological effects of CLU are complex and circumstance-dependent. Experimental investigation conducted on mice showed that intraventricular apolipoprotein ApoJ infusion acts protectively in traumatic brain injury by suppressing the inflammatory response (glial activation, cytokine expression), blood–brain barrier disruption, and cerebral edema (24). Taking into account the fact that the CLU concentrations are affected in various neurological diseases, we have hypothesized that brain ischemia-reperfusion injury after CEA can also change the concentration of this molecule. This change may be due to the previously described neuroprotective function of CLU against intracellular toxicity within the central nervous system.

The objective of our study was to report the changes in serum CLU concentrations of patients undergoing CEA.

## Materials and methods

The patients were admitted in the Department of Vascular Surgery and Angiology, Medical University of Lublin, Poland, and were scheduled to undergo CEA due to internal carotid artery stenosis. Based on Doppler studies, candidates were qualified for the CEA procedure as determined by the guidelines set forth by the European Society of Vascular Surgery. Patients with severe carotid artery stenosis were identified using criteria established by NASCET (North American Symptomatic Carotid Endarterectomy Trial) (25,26). The study involved 25 patients aged from 55 to 83 years with a mean age of 69 years. The degree of the internal carotid artery stenosis ranged from 60% to 92%. Neurological examination was performed by a neurologist prior to and after CEA. In this neurological study there were no deviations from the normal. The demographic information and pertinent past medical histories of patients are summarized in Table I.

**Table I.** Characteristics of patients.

Patient ID	Sex	Age	Carotid artery stenosis		Past medical history: stroke and TIA	Symptoms	Other diseases
			Location	%			
1	F	72	L	90	TIA	Transient hemiparesthesia right	Arterial hypertension
2	M	74	L	92	None	None	None
3	M	76	L	90	None	None	None
4	M	67	R	80	TIA	Transient hemiparesis left	Chronic obstructive pulmonary disease
5	F	70	L	60	TIA	Vertigo	Arterial hypertension, diabetes, ischemic heart disease
6	M	67	R	87	Hemorrhagic stroke	None	None
7	M	70	L	75	None	None	None
8	F	74	R	85	None	None	Arterial hypertension
9	F	65	R	82	2 ischemic strokes	Hemiparesis right	Arterial hypertension
10	M	69	L	80	None	None	None
11	F	66	R	79	TIA	Transient hemiparesthesia left	Arterial hypertension
12	M	77	R	88	None	None	None
13	M	67	R	80	TIA, ischemic stroke	Hemiparesis left	Arterial hypertension
14	F	67	L	85	TIA	Transient hemiparesis right	Diabetes
15	F	78	R	80	TIA	Transient hemiparesthesia left	None
16	F	70	L	90	TIA, ischemic stroke	Transient hemiparesis right	None
17	M	56	R	84	None	None	Metabolic syndrome, ischemic heart disease, hypothyreosis
18	F	68	L	90	None	None	None
19	F	62	L	90	TIA	Transient amaurosis in left eye	None
20	M	73	R	90	None	None	None
21	M	62	R	89	None	None	Arterial hypertension
22	M	83	L	86	Ischemic stroke	Aphasia, hemiparesis right	Arterial hypertension, atrial fibrillation
23	F	55	R	79	None	None	None
24	F	78	R	70	TIA	Transient amaurosis in right eye	Arterial hypertension, diabetes
25	M	66	L	85	None	None	Arterial hypertension, diabetes, Parkinson’s syndrome

F = female; L = left; M = male; R = right; TIA = transient ischemic attack.

Serum samples were taken from the antecubital vein of patients at three different times: within 24 hours preoperatively to CEA, 12 hours postoperatively, and 48 hours postoperatively. Serum CLU concentrations were measured using a commercially available enzyme-linked immunosorbent assay (CircuLex Human Clusterin/Apo-J ELISA Kit, CycLex Co., Ltd, Nagano, Japan).

Statistical analysis was performed using STATISTICA, version 12 software (StatSoft, Inc., Poland). The Mann–Whitney and Friedman tests were used to measure the differences between groups of patients. Non-parametric methods were used because the data were not normally distributed. The CLU concentration was measured in ng/mL. The level of statistical significance was *P* < 0.05.

The study was approved by the Ethics Committee of Medical University in Lublin (KE-0254/218/2014).

## Results

**Figure 1.**
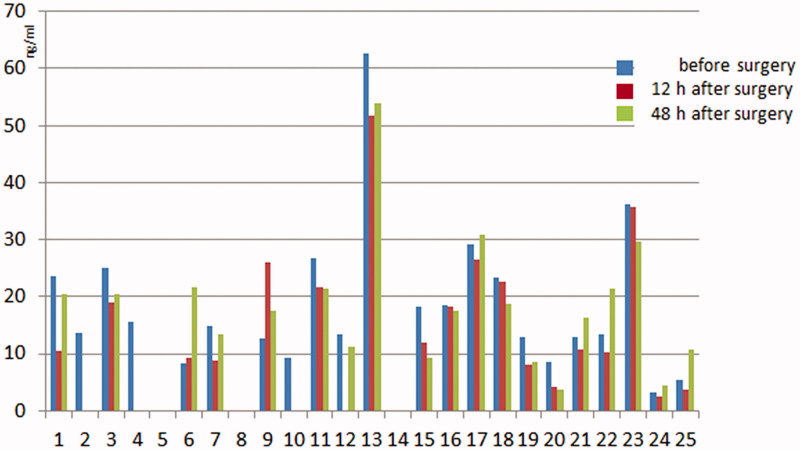
Serum CLU concentrations in patients.

Serum CLU concentrations in patients and a comparative analysis are presented in Figure 1 and Table II.

**Table II.** Serum CLU concentrations in patients.

Group of patients	*n*	Median (IQR) [ng/mL]			Comparison
		Before surgery [A]	12 h after CEA [B]	48 h after CEA [C]	
Total	25	13.43 (8.65, 23.32)	9.30 (0, 18.96)	16.31 (4.41, 21.42)	[A-B-C] *P* = 0.001^a^
Males	13	13.61 (9.39, 25.05)	8.73 (0, 10.85)	14.84 (7.25, 21.59)	[A] *P* = 0.58 [B] *P* = 0.53 [C] *P* = 0.60
Females	12	15.61 (1.63, 23.43)	11.24 (1.21, 22.14)	17.53 (4.41, 20.38)	
Younger (≤69 years)	13	13.00 (9.39, 26.81)	10.85 (3.66, 26.02)	18.18 (9.64, 25.72)	[A] *P* = 0.56 [B] *P* = 0.14 [C] *P* = 0.20
Older (>69 years)	12	13.52 (5.95, 18.36)	6.52 (0, 11.24)	11.29 (3.80, 20.38)	
Symptomatic	12	14.47 (8.02, 21.02)	10.41 (1.21, 20.03)	17.57 (8.59, 21.42)	[A] *P* = 0.93 [B] *P* = 0.76 [C] *P* = 0.70
Asymptomatic	13	13.43 (8.65, 23.32)	8.73 (0, 18.96)	13.36 (3.80, 20.41)	
Left artery stenosis	12	13.49 (7.44, 20.91)	8.45 (0, 14.40)	13.36 (0, 20.38)	[A] *P* = 0.64 [B] *P* = 0.26 [C] *P* = 0.32
Right artery stenosis	13	13.43 (8.65, 26.81)	10.85 (2.42, 26.02)	16.92 (6.90, 25.72)	
Artery stenosis ≤85%	14	13.84 (3.26, 26.81)	6.19 (0, 26.02)	12.03 (2.20, 25.58)	[A] *P* = 0.68 [B] *P* = 0.93 [C] *P* = 0.75
Artery stenosis >85%	11	13.43 (12.92, 23.32)	10.34 (4.32, 18.31)	17.60 (8.59, 20.41)	

a Statistically significant.

IQR = interquartile range.

Data from the study revealed that the serum CLU concentration initially decreased 12 hours after CEA when compared to concentration preoperatively, and then increased 48 hours postoperatively. The Friedman test indicated there were differences in serum CLU concentrations between all three recorded measurements (*P* < 0.05).

There was no difference in serum CLU concentrations in three measurements between males and females (*P* > 0.05). The difference in serum CLU concentrations between younger (≤69 years) and older (>69 years) patients in three measurements was not significant (*P* > 0.05).

There was no difference in serum CLU concentrations between preoperative symptomatic and asymptomatic patients (*P* > 0.05).

The difference in serum CLU concentrations between the left and right carotid artery stenosis in three measurements was not significant (*P* > 0.05). There was no difference between the groups of patients divided according to degree of carotid artery stenosis (*P* > 0.05). There was no significant difference between the degree of carotid artery stenosis in patients dependent on their age (*P* = 0.78).

## Discussion

Our study revealed that serum CLU concentrations are affected following CEA. CLU concentrations were decreased in the earliest period postoperatively. It has been previously hypothesized that ischemia-reperfusion injury may play a crucial role in brain damage following CEA. Therefore, alterations in CLU concentrations in the serum of patients may be reflective of the important function of this glycoprotein during central nervous system injury. Studies conducted on animal models support the neuroprotective role of CLU in early ischemic events, and have demonstrated that this glycoprotein plays a central role in the remodeling of ischemic damage (27). This study further suggests that CLU may also participate in ischemia-reperfusion injury after CEA. Data from current literature indicate that CLU concentrations may be affected in the CSF and plasma of patients during the pathogenesis of different neurological diseases and may also play a role as a neurological biomarker. Changes in the serum and CSF concentration of CLU are also present in patients with cerebrovascular diseases.

According to Wąsik et al. (28), inflammation following subarachnoid hemorrhage (SAH) involves numerous mediators with biomarker properties. These authors aimed to clarify the status of CLU in SAH. They observed that, following SAH, mean CSF CLU concentration decreased after 5–7 days, and then increased 10–14 days later. However, the CLU concentration failed to differentiate between good and poor prognosis at 0–3 days and 10–14 days after SAH, but significantly higher levels of CSF CLU were found 5–7 days after SAH in patients with good outcome. Thus, SAH was associated with a significant decrease in CSF CLU in their patients. However, there were no significant differences between CLU concentration in CSF at three time points (0–3 days after SAH versus 5–7 days after SAH versus 10–14 days after SAH). According to the authors, decreased CLU concentrations might be explained by the immediate binding of CLU to proteins and lipids from the extravasated blood. Moreover, the fact that the significant difference in CLU levels between patients with good and poor outcomes was observed only on days 5–7 after SAH might indicate that patients with good outcome more rapidly induce synthesis and secretion of CLU into the CSF. Similarly, the initial decrease in the serum CLU concentrations seen in our study after CEA may be due to the inclusion of this molecule in the mechanisms of nervous system protection after ischemia-reperfusion injury.

Furthermore, Song et al. (29) measured serum CLU in acute ischemic stroke patients within 24 hours of stroke onset and observed significantly higher serum CLU concentrations in stroke patients than in healthy controls. The serum values of CLU were also positively correlated with the NIH Stroke Scale (NIHSS) scores, the time interval after stroke onset, as well as major stroke risk factors associated with lipid profile. The authors concluded that elevated serum CLU concentration is independently associated with ischemic stroke and may serve as peripheral biomarker to aid in clinical assessment of ischemic stroke and its severity. According to the authors, the results from their study are consistent with previous findings suggesting that ischemic insult is associated with an overall downregulation of beneficial neurotrophic molecules and an upregulation of inflammatory signaling proteins and cytoskeletal components.

Additionally, according to Yu et al. (30), the CLU concentration was decreased in the CSF of human models diagnosed with epilepsy. Therefore, measurement of CLU concentrations in the CSF may be helpful in generating differential diagnosis of neurodegenerative disorders (31). It has been observed that CLU concentration was also decreased in the plasma of patients diagnosed with Alzheimer’s Disease when compared to control populations (32).

The aim of the study conducted by Maskanakis et al. (33) was to clarify the predictive value of ApoJ in internal carotid artery restenosis following CEA. The serum ApoJ levels of 100 patients were examined; 56 patients who underwent CEA constituted the vascular group (VG), and 44 patients constituted the control group (CG). ApoJ samples were obtained preoperatively, 24 h after the surgical procedure, and at 1, 6, and 12 months thereafter during the follow-up period. The preoperative differences in ApoJ levels between the CG and VG were statistically significant; the mean values were higher in the VG. In the VG, the serum ApoJ concentrations were higher at postoperative day 1 compared to preoperative levels, and were decreased at 1, 6, and 12 months postoperatively. Meanwhile, the ApoJ levels of patients in the CG remained unchanged. Further subdivision of the VG into patients with or without restenosis revealed that restenotic patients presented with significantly higher mean ApoJ values than those that were non-restenotic in the VG patients. According to the authors, ApoJ seems to be an important predictor for carotid restenosis at 6 and 12 months postoperatively. In our study there was no difference in serum CLU concentrations depending on the degree of artery stenosis.

Previously we assessed different markers of brain damage after CEA, and we obtained data similar to our present study. Our earlier study showed that microtubule-associated protein tau (MAPt) and myelin basic protein (MBP) concentrations were also significantly decreased 12 hours after CEA when compared to the level before the surgery. Furthermore, levels of these parameters were also normalized 48 hours after CEA. MAPt and MBP levels showed a characteristic time curve in patients who underwent CEA and did not experience any neurological deficit in the perioperative period. We concluded that possible alterations of this time curve may potentially be an index of neurological event occurrence (34). In our next study we observed that serum carnosine dipeptidase 1 (CNDP1) and ubiquitin C-terminal hydrolase L1 (UCHL1) concentrations were also significantly decreased 12 hours after CEA when compared to these concentrations before the surgery. Just as with the previous studies, these enzyme concentrations also normalized 48 h after CEA. Thus, we concluded that CEA significantly affects serum CNDP1 and UCHL1 concentrations, and therefore these enzyme concentrations seem to reflect brain ischemia resulting from severe internal carotid artery stenosis in patients undergoing CEA. However, these observed changes in serum CNDP1 and UCHL1 concentrations do not necessarily warrant a change in recommendations concerning the use of CEA in patients with high-grade internal carotid artery stenosis (35).

As previously mentioned, ischemia-reperfusion injury can play a critical role following CEA. Liu et al. (36) revealed that CLU reduces cold ischemia-reperfusion injury in heart transplantation through regulation of NF-kB signaling and Bax/Bcl-xL expression. According to the authors, CLU has a protective effect caused by inhibition of cell apoptosis/death and reducing the pre-inflammatory response. Li et al. (37) also observed reduction of cold ischemia-reperfusion injury by using graft samples expressing CLU during heart transplantation. Additionally, other authors demonstrated promotion of cell proliferation by CLU in the renal tissue repair phase after ischemia-reperfusion injury (38). Moreover, it was revealed that loss of CLU expression worsened renal ischemia-reperfusion injury (39). These studies clearly provide evidence of the protective effects of CLU during the ischemia-reperfusion injury.

In summary, data from our present study indicate that serum CLU concentrations are affected following CEA. CLU produced in the brain can be measured in the serum secondary to blood–brain barrier rupture possibly following ischemia-reperfusion injury. As previously mentioned, the initial reduction of serum CLU concentrations in our study may indicate the involvement of this molecule in the central nervous system protectant mechanisms against ischemia-reperfusion injury. However, it should not be excluded that a significantly decreased serum CLU concentration during the earliest period (12 hours after CEA) of ischemia-reperfusion injury may disrupt the cytoprotective effect of this protein. When compared to the preoperative serum CLU concentrations, we hypothesize that the increase in serum CLU levels 48 hours postoperatively may indicate CLU’s neuroprotective function on the brain after CEA, and thus might explain the absence of neurological complications in our examined patients. Therefore, it is possible that serum CLU concentrations can be dependent upon brain ischemia-reperfusion injury following CEA. As in our previous studies, the CLU concentration also showed a characteristic time curve in our patients without neurological deficit in the perioperative period. Thus, any deviant alteration of this time curve might be a reflection of potential neurological complication.

Our study presents new data on the CLU after CEA. However, the study has some limitations, and therefore it cannot be excluded that additional factors, e.g. anesthesia or/and physical injury caused by surgery, may influence CLU concentrations. Another such limitation is also the low power of the study to reveal group differences.
